# Defining effective strategies to prevent post-traumatic stress in healthcare emergency workers facing the COVID-19 pandemic in Italy

**DOI:** 10.1017/S1092852920001637

**Published:** 2020-07-14

**Authors:** Claudia Carmassi, Giancarlo Cerveri, Eric Bui, Camilla Gesi, Liliana Dell’Osso

**Affiliations:** 1 Department of Clinical and Experimental Medicine, University of Pisa, Pisa, Italy; 2 Department of Mental Health and Addiction, ASST Lodi, Lodi, Italy; 3 Massachusetts General Hospital, Boston, Massachusetts, USA; 4 Harvard Medical School, Boston, Massachusetts, USA; 5 Department of Mental Health and Addiction, ASST Fatebenefratelli-Sacco, Milan

Italy, May 19th. As we write, the end of the COVID-19 outbreak is finally in view; however, its psychological impact on healthcare workers (HCWs) remains an urgent question. Italy was the first European Country to face the pandemic that started in Codogno (Lodi), declared “red zone” and isolated by February 20th 2020. To date, the number of confirmed COVID-19 cases exceeds 215 000, with a death toll exceeding 30 000, over 23 000 HCWs being infected and 161 of them—130 physicians and 31 nurses—losing their life to complications of the infection. Because HCWs facing the COVID-19 outbreak were exposed to extreme and prolonged work-related stress with a high risk of exhaustion, burnout and post-traumatic stress reactions, the COVID-19 crisis was defined as the “*9/11 of health care systems.*”

Since the introduction of the Diagnostic and Statistical Manual for Mental Disorders—5th edition (DSM-5), work-related repeated or extreme exposure to aversive details of traumatic events may qualify for a trauma. In line with this, recent literature on burnout and post-traumatic stress disorder (PTSD) among first responders and HCWs, highlighted specific risk factors such as the frequent unpredictability of daily work cases and perceived expectations from patients and their families in critical cases/situations.^1^ ^,^^2^ While the Italian health care system was weakened by years and years of budgetary cuts, the COVID-19 outbreak amplified the chronic difficulties, leading to increased risk for negative mental health outcomes among HCWs.

First, typical pre-existing risk factors for PTSD were frequently present among Italian HCWs, including female gender and young to middle age with young children. Second, specific characteristics of COVID-19 added further burden: rapidly increased flow of critical patients forcing physicians to make extremely difficult decision tainted with pervasive helplessness; clinical presentation characterized by severe distress, rapidly worsening dyspnea with insidious and unpredictable course, requiring constant medical updating with worldwide emerging multiple clinical manifestations and treatment of the COVID-19 infection increasing distress at the time of patient’s death; constant need for complete isolation during patient care, both for the patient and the providers, given the extremely high contamination risk, and the shortage of protective personal equipment that lead to fear, anger, and resentment against the authorities. Third, the systemic impact of the disease and its social repercussions also included: rapid and sometimes chaotic reorganization of services and logistical challenges for recruiting new emergency departments and intensive care unit (ICU) personnel, both to managing the increasing number of patients and to substitute HCWs becoming ill; introducing unskilled HCWs in highly specialized units, leading to feelings of frustration, isolation, hopelessness, and irritability; lack of social support at work; the need to work with new colleagues or redeployed strangers; lack of social support at home with schools closures and children confined at home; fear of infecting family members leading to further isolation.

Taken together, there was an urgent need to efficiently provide mental health support to first-line HCWs early in the outbreak, to promote resilience and mitigate long-term effects of exposure to multiple COVID-related stressors. Similarly, to Chinese hospital,^3^ ^,^^4^ Codogno Hospital (Lodi) rapidly delineated and deployed mental health support to first-line HCWs in collaboration with researchers at the Pisa University Hospital (Psychiatric Clinic), based on their expertise developed in most recent years, in particular during the L'Aquila earthquake.^5^

A psychological first management service for emergency personnel was set up in Codogno (Lodi), with a psychiatrist and a psychologist, to address HCW’s hyperarousal, irritability, trouble sleeping, psychological distress, and unwillingness to rest. As the pandemic spread across Italy, a nationwide “red zone” was declared on March 10th, and all hospitals reorganized to face the COVID-19 crisis leveraging the 2 weeks of experience of the northern regions. HCWs were however reluctant to access the psychological interventions, requiring instead support for patient management. They reported difficulties in facing the needs of their patients’ who were in total isolation, as they were their only means of communication with family members and the outside world. Consistently, they reported the emotional burden of having to convey by phone in an expedited manner, important clinical information—including a death—to family members. This was particularly reported in Pisa, where an open-door ICU program was successfully set up and the COVID-19 emergency determined a radically reversed approach. Technological support to communicate with families was helpful to alleviate the impact of isolation and loneliness for patients, and to support HCWs. In parallel, defining specific times to communicate with patients’ families allowed HCWs to carve out some time to cope with the burden of communicating with families, most of whom reported more than one affected or dead relative, particularly in most affected area. Fourth, despite the nationwide support to HCWs portrayed by the media, some reported perceived stigmatization when going back home, as people appeared scared that they may be contagious.

As the number of patients decreased over time, psychological support programs for HCWs received increasing number of requests, with HCWs being more and more willing to report psychological distress. As units were organized and guidelines for patient management and treatment defined, HCWs shifted their attention from their patients’ needs to their own needs for emotional support. Thus, a screening program for acute stress, anxiety and depressive symptoms, and their impact on work and social functioning was developed and implemented, both in Codogno and Pisa, in order to follow-up subjects for PTSD risk and monitor support impact. Supporting HCW’s mental health requires diversified intervention strategies, which should be delivered both in the immediate aftermath of the COVID-19 crisis and in the medium and long term, and specifically in the so called “second phase,” as we should focus our attention on those who helped the country to face this unprecedented health emergency. Lessons learned from these psychological interventions will inform government and authorities on how to respond to future unexpected infectious disease outbreaks.
